# Empathy in Toddlers: The Role of Emotion Regulation, Language Ability, and Maternal Emotion Socialization Style

**DOI:** 10.3389/fpsyg.2020.586862

**Published:** 2020-10-20

**Authors:** Veronica Ornaghi, Elisabetta Conte, Ilaria Grazzani

**Affiliations:** “Riccardo Massa” Department of Human Sciences for Education, University of Milano-Bicocca, Milan, Italy

**Keywords:** empathy, emotion regulation, vocabulary, maternal emotion socialization style, toddlers

## Abstract

We investigated, through a cross-sectional study, whether and to what extent toddlers’ empathy is associated with a set of individual and family factors known to foster positive social skills in early childhood: children’s own emotion regulation, language ability, and maternal emotion socialization style. Participants were 320 toddlers (*M*_age_ = 28.8 months; *SD* = 3.55) and their mothers. The children came from middle-SES families and were recruited at 34 infant-toddler centers. We used parent-report measures to assess the toddlers’ competences and a self-report questionnaire to evaluate maternal emotion socialization style (coaching vs. dismissing). Toddlers’ empathic responses, as reported by their mothers, were positively and significantly correlated, respectively, with their positive emotion regulation, language skills, and maternal emotion-coaching style. Stepwise regression analysis revealed that emotion regulation and maternal emotion-coaching style contributed to explaining variance in toddlers’ empathy, after controlling for the effects of children’s age and language ability. Moderation analysis showed that emotion regulation skills did not moderate the relationship between maternal emotion-coaching style and children’s empathy. We discuss the implications of these findings.

## Introduction

Empathy is made up of as a set of processes enabling an individual to attend to, understand, and attune to the feelings, bodies, and minds of others by directly observing or imagining their emotional states (Hollan, 2012; Preston and Hofelich, 2012). In the course of toddlerhood, children’s response to distress in others shifts from overwhelming personal distress to a more other-oriented empathic reaction that often prompts precocious prosocial behaviors. Empathic competence functions as a protective factor against non-adaptive responses from the early years through childhood and adolescence (Zahn-Waxler and Van Hulle, 2012; Blandon and Scrimgeour, 2015). It entails a shift from concern for self to concern for others, and develops alongside gains in self-other differentiation, perspective taking, and emotion regulation (Knafo et al., 2008; Conte et al., 2019). Both children’s early functioning, especially their emotion regulation of arousal, and the environmental context (e.g., maternal emotion socialization practices) where these skills develop, can foster positive behaviors or, on the contrary, contribute to behavioral difficulties.

The aim of the present study was to conduct an in-depth examination of the relations between toddlers’ empathy and both intra-individual (emotion regulation and language ability) and family factors (maternal emotion socialization style). Although all these factors have been individually found to foster the development of positive social skills (Denham, 2007), their contributions to explaining variance in empathic responses during early childhood remain to be examined within a comprehensive research design. We innovatively chose to investigate this set of relations in toddlerhood, a period that sees strong development in all the competences and behaviors under study. We now take an in-depth look at these factors in order to build up a rationale for the present study.

### The Development of Empathy in Early Childhood

Empathy is a multifaceted construct comprising an affective, a cognitive, and a behavioral component (Geangu, 2015; Telle and Pfister, 2016). The affective component entails empathic concern (i.e., the ability to feel, share, and respond to others’ emotional experiences); the cognitive component consists in perspective-taking abilities (i.e., understanding, and attributing others with, mental states and viewpoints; Lonigro et al., 2014), while the behavioral or external component implies prosocial conducts (Bensalah et al., 2016).

Being empathic toward others requires some awareness of the difference between self and others. Hoffman (2008) identified three levels of empathy in early childhood, which develop sequentially and progressively. The first level, emotional contagion, is an innate, automatic, and non-modulated response to others’ emotions, due to an immature sense of self whereby the child is not yet able to perceive itself as a separate entity (Roth-Hanania et al., 2011; Bischof-Köhler, 2012). It consists of the synchronized expression of emotion via vocalizations and non-verbal channels (posture, movements, facial expressivity, etc.) and is observable from the first year of life, as for example when a newborn’s crying triggers another baby’s crying. The second level develops at around 1 year of age and involves paying attention to others’ feelings. Having acquired a greater ability to differentiate between themselves and others, children at this age can attend to another person’s feelings, recognizing that a given emotion is being experienced by another, and experiencing a similar emotion themselves. Hence, at this age, children begin to adopt strategies aimed at regulating their own emotions by displaying concern for another person (Montroy et al., 2016). Finally, the third level of empathy is acquired at around 2 years of age when children’s greater understanding of others’ mental states and situations motivates them to engage in prosocial behaviors (Eisenberg et al., 2015; Geangu, 2015).

This developmental sequence suggests that empathy may be observed from a very young age. However, differences in children’s empathy are shaped by multiple factors, such as child emotion regulation and verbal ability, and mothers’ emotion socialization style. In the next sections of the paper, we focus on each of these variables in turn and its associations with empathic responses, with a view to exploring the contribution of these individual and family factors to variance in empathy.

### The Relationship Between Emotion Regulation and Empathy

Emotion regulation is the ability to handle, modulate, inhibit, and enhance emotional expressions and responses by deploying appropriate and effective strategies (Eisenberg and Spinrad, 2004; Gross, 2007; Denham et al., 2012). It may rely on internal processes (e.g., management of physiological responses, attention shifting) or external factors (e.g., asking other people for help) and may already be observed in the first months of life (Trevarthen, 2011), for example, when newborns and infants in stressful situations display primitive self-soothing mechanisms such as sucking or shifting their gaze (Calkins and Leerkes, 2011). Around the end of their first year, children realize that they can ask for caregivers’ support in regulating their emotional states and seeking to manage their feelings. Then, at about 2 years, alongside the development of attention control mechanisms, toddlers begin to identify active and flexible strategies for regulating emotion under a variety of circumstances. For example, when they experience stressful events, they may try to distract themselves with other stimuli, complain about perceived unfair treatment, or modify the situation itself by seeking an effective solution (Kurki et al., 2017).

There is evidence that emotion regulation is significantly related to empathy in toddlerhood. Several studies have examined how the ability to control emotion arousal can affect empathy in toddlerhood and the preschool years. Peterson et al. (2018) showed that better emotion regulation in 9-month-old infants predicted higher levels of empathy at 2 years of age. Panfile and Laible (2012) asked mothers to complete questionnaires about their children when they were exactly 36 months. Path analyses showed that children’s emotion regulation ability is significantly associated with their empathic responses. Similar results were obtained in studies with older children. For example, Laible et al. (2014) carried out a longitudinal study showing that moderate to high levels of regulation in preschoolers predicted a higher level of empathy later in childhood. Conversely, poor regulation abilities were associated with fewer empathic responses. In sum, it appears that possessing emotion regulation skills allows children to redirect their attention from their own distress to that of others, a key prerequisite for empathic responses.

### Language Abilities and Empathy

While the association between language acquisition and empathy has received scant attention in the literature, recent outcomes suggest that children’s language skills may play both a direct and an indirect role in their empathic responses and behaviors (e.g., Ensor and Hughes, 2005; Ornaghi et al., 2015; Girard et al., 2017; Ornaghi et al., 2017; Conte et al., 2018). For example, Rhee et al. (2013) conducted a longitudinal study with twins at 14, 20, 24, and 36 months, reporting that more advanced language ability predicted greater concern for others and less disregard for others, even after controlling for cognitive abilities. Interestingly, these associations were maintained at different ages, showing that they were stable over time. In a similar longitudinal study with children at 2, 3, and 4 years, Ensor et al. (2011) found that verbal ability and prosocial orientation – a composite measure of empathic concern and prosocial action – were strongly and significantly associated with one another at all three time points.

The key role of language in explaining empathic responses was borne out even by training studies which pointed out the indirect effects of language on empathic conducts. For instance, Grazzani et al. (2016a) conducted a conversation-based training program in early education centers with the aim of improving toddlers’ social and emotional skills. The intervention was found to significantly improve children’s empathy scores – and specifically their ability to pay attention to the feelings of others. Furthermore, Ornaghi et al. (2017) tested the effects of an emotion-based intervention conducted by trained day-care teachers on toddlers’ prosocial behaviors. Children were read brief stories written by the research team; each story was focused on a specific basic emotion (such as fear, anger, happiness, and sadness). Then children in the experimental group were involved in conversations about the target emotion of the story. Findings showed that they outperformed the control group not only in terms of their emotional competence, but also in terms of the frequency with which they displayed empathic and prosocial responses toward their peers.

### Maternal Emotion Socialization Styles and Children’s Emotion Regulation and Empathy

Children’s development of social and emotional skills, especially early in life, is widely reported to be influenced by the family environment, quality of early parenting, and emotion-related parenting practices (Blandon and Scrimgeour, 2015; Zeytinoglu et al., 2017). Emotion socialization consists of a set of cultural practices, that begin in the family settings and extend outward, conveying the modes and strategies to express and regulate emotions within social interactions (Denham, 2007). Children who are exposed to positive emotional expressivity, discourse about emotions, and positive acceptance of emotional displays in the home exhibit higher levels of social-emotional competence than do children whose parents avoid focusing on emotional experience (Morris et al., 2007; Brophy-Herb et al., 2011; Gross et al., 2015; Ornaghi et al., 2019). It is worth noting that the socialization of emotion is a multifaceted and complex process, not a simple transfer of skills from parents to children. Eisenberg et al. (1998) proposed a heuristic model where parental emotion socialization affects children’s social-emotional competences both directly and indirectly via the mediation of child’s arousal. Furthermore, other variables, such as parental characteristics, are likely to influence emotion socialization and to moderate the association between these practices and children’s emotional and behavioral outcomes (Eisenberg et al., 1998). A well-known study by Gottman et al. (1997) shows that parents’ individual beliefs and feelings about their own and their children’s emotions shape their reactions to their children’s expression of emotion, and influence how they socialize their children’s understanding and expression of emotional experience. Similarly, it is well documented that parents’ emotion regulation skills and related emotion socialization practices influence children’s emotion regulation development (Rutherford et al., 2015; Li et al., 2019).

With regard to parental approaches to interaction, two main emotion socialization styles have been described in the literature: the emotion-coaching and emotion-dismissing styles (Gottman et al., 1997). The emotion-coaching style is typical of parents who are aware of their own and their children’s emotions, discuss emotions and feelings with them, view their children’s negative emotions as opportunities to promote their emotional competence, and use emotional situations constructively. On the contrary, parents who display an emotion-dismissing style suffer from a lack of awareness of their own and their children’s emotions, and react negatively to expressed emotions – especially negative ones – for example by ignoring, minimizing, or disapproving of them (e.g., Lunkenheimer et al., 2007).

There is evidence that these styles greatly influence social and emotional learning both in preschoolers and older children (Dunsmore and Karn, 2004; Morris et al., 2007; Dunsmore et al., 2012). Indeed, children whose parents draw on a predominantly emotion-coaching style display more advanced emotion knowledge and emotion regulation, greater social adjustment, more positive peer relations, greater academic success, and higher self-esteem than children whose parents implement an emotion-dismissing style (e.g., Gottman et al., 1997; Legacé-Séguin and Coplan, 2005; Ornaghi et al., 2019).

There is also empirical evidence that maternal emotion socialization influences children’s development of empathy. Specifically, children whose mothers accept and positively react to their emotions are more empathic, while children whose mothers discourage emotion expression – thereby denying them the opportunity to learn about their own and others’ feelings – tend to display lower levels of empathy-related responding (Eisenberg et al., 2011). Brophy-Herb et al. (2011) investigated the impact of maternal emotion socialization and contingent responsiveness on toddlers’ social-emotional competence in a low-income sample, finding that maternal emotion coaching directly and indirectly (via maternal responsiveness) predicted child competences such as compliance, empathy, mastery, and motivation. In addition, Taylor et al. (2013) found that mothers’ emotion socialization practices at 18 months predicted changes in empathy across the early years, which in turn predicted later prosocial behavior with peers.

### The Present Study

The core aim of this study was to investigate the extent to which a set of individual and family factors are associated with toddlers’ empathic responses. More specifically, we first set out to verify the association between toddlers’ empathy and their emotion regulation, language ability, and maternal emotion socialization style (coaching vs. dismissing). In light of the literature just reviewed, we expected to find significant correlations among the study variables. Second, we aimed at exploring the relative contributions of emotion regulation and maternal emotion socialization style to explaining variance in toddlers’ levels of empathy, while controlling for their age and language ability. In line with previous studies, we expected that empathy would be crucially influenced by both a maternal emotion-coaching style (e.g., Eisenberg et al., 2011; Taylor et al., 2013) and toddlers’ emotion regulation skills (e.g., Panfile and Laible, 2012; Peterson et al., 2018).

## Materials and Methods

### Participants

The research sample comprised 320 toddlers (156 girls) with a mean age of 28.8 months (*SD* = 3.55; range: 24–36) and their mothers. Participants were recruited at 34 infant-toddler centers in Northern Italy. All toddlers were native Italian speakers whose linguistic and cognitive development fell within the standards for their age group and came from middle-SES families. Mothers’ mean age was 35.71 years (*SD* = 5.12; age range: 20–48). The majority held a high school diploma or university degree (89.4%) and were either white-collar employees or self-employed professionals (78.4%). Other mothers were manual workers (18.6%), while the remainder were unemployed (3.0%). In addition, 38.9% of the toddlers were only children, 46.6% had one sibling, 10.9% had two siblings, and the remaining 3.6% three or more siblings.

### Measures and Procedure

The study was approved by the Ethics Committee of the University of Milano-Bicocca. The researchers intervened in the meeting that the services organized at the beginning of the school year with all parents. They brief them about the general aim of the study and parents provided informed consent for themselves and their children. Participation in the study was 98% of parents. The mothers were given an envelope containing four questionnaires, presented in counterbalanced order, assessing their children’s empathic responses, vocabulary and emotion regulation, and their own emotion socialization style. They were asked to complete the questionnaire at home and to return them within 1 week.

Missing data were <1% of the overall data and were treated by substituting them with the row average score (Graham et al., 2012).

#### Empathy Questionnaire – I13

The *Empathy Questionnaire – I13* (EmQue-I13; Grazzani et al., 2017) is the Italian validated version of the Empathy Questionnaire by Rieffe et al. (2010). It is a parent-report measure composed of 13 items assessing empathy-related behaviors in young children. Items divide into a three-factor structure measuring the dimensions of *Contagion* (4 items; sample item: “My child also needs to be comforted when another child is in pain”), *Attention to others’ feelings* (5 items; sample item: “When an adult gets angry with another child, my child watches attentively”), and *Prosocial actions* (4 items; sample item: “When another child gets upset, my child tries to cheer him/her up”). Respondents are asked to rate the degree to which each item would have applied to their child over the previous 2 months on a 5-point-scale (1 = never, 2 = rarely, 3 = sometimes, 4 = often, 5 = always). Higher scores reflect higher levels of the corresponding behaviors. Reliability values for the global questionnaire and the three subscales were satisfactory, EmQue: α = 0.77; Contagion subscale: α = 0.65; Attention to Others’ Feelings subscale: α = 0.64; Prosocial Actions subscale: α = 0.80 (α = 0.73, 0.74, and 0.80, respectively, for the three subscales in the validated original instrument; see Grazzani et al., 2017). Mean inter-item correlations in the present study were *r* = 0.32 for Contagion, 0.30 for Attention to Other’s Feelings, and 0.50 for Prosocial Actions, in line with the original instrument (*r* = 0.19, 0.26, and 0.41, respectively; Rieffe et al., 2010).

#### McArthur-Bates Communicative Development Inventories

*McArthur-Bates Communicative Development Inventories* (Fenson et al., 2000). Mothers completed the short Italian version of the questionnaire (Caselli et al., 2007), a standardized and validated instrument that assesses language abilities based on maternal ratings of children between 18 and 36 months of age. The instrument evaluates the child’s word production (vocabulary), ability to formulate phrases of several words (complexity), and pragmatic abilities, including pointing, making gestures, pretending (pragmatics). In the current study, mothers were asked to complete the vocabulary section only for ease of administration and to avoid respondent fatigue. The standard scoring procedures were applied. Participants’ scores for the vocabulary section ranged from 0 to 100. The internal consistency coefficient computed for this scale was Guttman’s λ 4 = 0.78.

#### Emotion Regulation Checklist

The *Emotion Regulation Checklist* (ERC; Molina et al., 2014). This is an adult-report scale assessing the dimensions of positive emotion regulation and negativity. We administered the Italian validated version of the instrument, whose 24 items describe aspects of children’s emotionality and regulation, such as affective lability, intensity, valence, flexibility, and situational appropriateness. The mothers were asked to evaluate the frequency of these behaviors on a 4-point Likert scale, from 1 (almost never) to 4 (almost always). Children received a separate score for each of two subscales: Emotion Regulation (8 items; score range: 8–32; sample item: “Displays appropriate negative emotions – anger, fear, frustration, distress – in response to hostile, aggressive and intrusive acts by peers”) and Lability/Negativity (16 items; score range: 16–64; sample item: “Responds angrily to limit setting by adults”). Higher scores on the Emotion Regulation scale indicate a greater capacity to manage and modulate one’s own emotional arousal, whereas higher scores in the Lability/Negativity scale indicate dysregulation, inflexibility, negative affect, and excessive emotional reactions. Internal consistency coefficients for the Emotion Regulation and Lability/Negativity scales were α = 0.72 and α = 0.65, respectively.

#### Maternal Emotional Style Questionnaire

The *Maternal Emotional Style Questionnaire* (MESQ, Legacé-Séguin and Coplan, 2005). This instrument assesses the maternal emotional behaviors produced in response to children’s emotion displays. Mothers are asked to express their level of agreement with each of the 14 items, on a 5-point Likert scale ranging from 1 (strongly disagree) to 5 (strongly agree). In the present study, we used the Italian version of the MESQ (Ciucci and Menesini, 2008). Each mother received two scores, one reflecting her Emotion-Coaching behaviors (seven items, score range: 7–35; sample item: “When my child is angry, I take some time to experience this feeling with him/her”) and one tapping into her Emotion-Dismissing behaviors (7 items, score range: 7–35; sample item: “When my child is angry, my goal is to make him/her stop”). Reliability coefficients for the Emotion-Coaching and Emotion-Dismissing scales were α = 0.70 and α = 0.77, respectively.

## Results

The results are divided into two sections. First, we report descriptive statistics for all measures and the zero-order correlations among study variables. Second, we present the outcomes of the regression analysis.

### Descriptive Statistics and Correlations

Data were normally distributed. The main statistical descriptives for all measures are presented in Table 1, and the zero-order correlations among variables are summarized in Table 2. In general, the correlation analysis revealed significant associations among the study variables. More specifically, children’s positive emotion regulation was found to be significantly correlated with language ability (*p* < 0.001). Also, it was negatively associated with maternal emotion-dismissing style (*p* < 0.001) and positively correlated with maternal emotion-coaching style (*p* = 0.03).

**TABLE 1:** Descriptives for the study variables.

	*M*	*SD*	Range	Skewness	Kurtosis
Age in months	28.792	3.549	24–36	–0.102	–0.875
Vocabulary	54.669	28.150	1–100	–0.114	–0.990
EmQue total score	37.865	6.047	20–54	–0.229	0.170
EmQue emotion contagion	8.884	2.676	4–16	0.169	–0.484
EmQue attention to others’ feelings	19.219	2.617	11–24	–0.390	–0.022
EmQue prosocial actions	9.755	3.027	4–18	0.015	–0.295
ERC positive emotion regulation	25.304	2.658	16–32	–0.364	0.365
ERC lability/negativity	28.791	5.832	18–56	0.917	–0.295
MESQ coaching style	27.804	3.742	15–35	–0.501	0.193
MESQ dismissing style	22.247	5.483	7–34	–0.163	–0.282

*EmQue, empathy questionnaire; ERC, emotion regulation checklist; MESQ, maternal emotional style questionnaire.*

**TABLE 2:** Zero-order correlations among the study variables.

	(1)	(2)	(3)	(4)	(5)	(6)	(7)	(8)	(9)	(10)	(11)
Age (1)	–	0.010	0.393***	0.087	0.042	–0.008	0.144*	0.053	0.160**	0.012	0.023
Gender (2)		–	0.143*	0.027	0.015	–0.040	0.074	–0.093	0.002	–0.030	–0.025
Vocabulary (3)			–	0.221**	0.111	0.164**	0.204***	–0.088	0.445***	0.070	−0.246**
EmQue total score (4)				–	0.693***	0.717***	0.765***	–0.033	0.403***	0.173**	0.053
EmQue emotion contagion (5)					–	0.264***	0.273***	0.035	0.159***	0.047	0.049
EmQue attention to others’ feelings (6)						–	0.333***	0.046	0.388***	0.191**	–0.008
EmQue prosocial actions (7)							–	–0.079	0.329***	0.138*	0.067
ERC lability/negativity (8)								–	−0.125*	0.024	0.162**
ERC positive emotion regulation (9)									–	0.117*	−0.212**
MESQ coaching style (10)										–	0.246**
MESQ dismissing style (11)											–

*^∗^*p* < 0.05; ^∗∗^*p* < 0.01; ^∗∗∗^*p* < 0.001. EmQue, empathy questionnaire; ERC, emotion regulation checklist; MESQ, maternal emotional style questionnaire.*

Toddlers’ empathy ratings were significantly correlated with their positive emotion regulation ability (*p* < 0.001) and vocabulary (*p* < 0.001), as well as with maternal emotion-coaching style (*p* = 0.002). With regard to the three subscales of the EmQue-I13, scores on both the Attention to Others’ Feelings and Prosocial Behavior subscales were significantly correlated with positive emotion regulation (*r* = 0.388; *p* < 0.001; *r* = 0.329; *p* < 0.001), vocabulary (*r* = 0.164; *p* = 0.004; *r* = 0.204; *p* < 0.001), and maternal emotion-coaching style (*r* = 0.191; *p* = 0.001; *r* = 0.138; *p* = 0.015), whereas scores on the Contagion subscale were only significantly correlated with positive emotion regulation (*r* = 0.159; *p* = 0.005). Finally, only scores on the Prosocial Behavior subscale were positively correlated with age (*r* = 0.144; *p* = 0.010).

### Regression Analyses

We conducted linear regression analysis to investigate whether and to what extent children’s emotion regulation skills, language ability, and maternal emotion-coaching style contributed to explaining toddlers’ empathy. Children’s gender, as well as maternal emotion-dismissing style, were not included in the regression model given that they were not significantly correlated with our target variable (see Table 3). Furthermore, in order to control possible source of covariance, ICC analysis was computed to test whether center membership was associated with variables under study. The result, ICC = 0.101 [CI 95% 0.083−0.185], showed that center membership explained a marginal portion of variance, not exceeding the recommended value. Consequently, the variable infant-toddler center was not included in the regression analysis (Heck et al., 2014).

**TABLE 3:** Regression outcomes (standardized beta weights) for the target variable empathy.

		β	*SE*	*t*	*p*
Step 1					
	Age	0.011	0.103	0.175	0.861
	Vocabulary	0.211	0.013	3.444	0.001
Step 2					
	Age	0.025	0.097	0.437	0.663
	Vocabulary	0.038	0.014	0.599	0.550
	Positive emotion regulation	0.385	0.136	6.548	< 0.001
Step 3					
	Age	0.035	0.096	0.612	0.541
	Vocabulary	0.027	0.013	0.432	0.666
	Positive emotion regulation	0.376	0.135	6.461	< 0.001
	Maternal emotion-coaching style	0.148	0.086	2.821	0.005

The regression model was designed to assess variance in empathy across three consecutive steps. First (Step 1), we investigated the role of age and language ability (vocabulary). Next (Step 2), we examined the influence of positive emotion regulation by entering it in the regression model. Finally, at Step 3, we explored the contribution of maternal emotion-coaching style. We assessed the regression model after each step by seeking statistically significant variations in the coefficient of determination (*R*^2^) and standardized beta weights (β). We also computed Cohen’s effect size for the model.

When children’s age and language ability were entered together at Step 1, the model was statistically significant, *F*(2,318) = 7.30, *p* = 0.001, and accounted for approximately 5% of explained variance. Only language ability explained variability in empathy scores (β = 0.211, *p* = 0.001). At Step 2, *F*(3,316) = 19.84, *p* < 0.001, the inclusion of toddlers’ positive emotion regulation led to a statistically significant increase of 11% in explained variance (*R*^2^ = 0.17). More specifically, positive emotion regulation (β = 0.385, *p* < 0.001) had a significant impact on empathy scores, whereas language was no longer significant. Finally, when maternal emotion-coaching style was entered into the regression model, *F*(4,314) = 7.96, *p* = 0.005, the explained variance increased by 2% (*R*^2^ = 0.19). In sum, both positive emotion regulation and maternal emotion-coaching style made a significant contribution to explaining variability in scores on the EmQue-I13, although emotion regulation (β = 0.376; *p* < 0.001) had a stronger impact than maternal emotion socialization (β = 0.150; *p* = 0.005).

### Moderation Analysis

Given the important role of emotion regulation in explaining toddlers’ empathic responses, we next assessed a model where positive emotion regulation was hypothesized to moderate the relationship between maternal emotion-coaching style and toddlers’ empathy. Since regression analyses revealed that children’s age and vocabulary had no statistically significant impact on empathy scores, they were not included in the model.

Moderation analysis was run using the SPSS PROCESS macro (Hayes, 2017). We tested a model with maternal emotion-coaching style as the predictor and toddlers’ emotion regulation as the moderator of the effects on empathy. The moderation analysis showed that the association between maternal emotion-coaching style and children’s empathy was not significantly moderated by children’s emotion regulation skills (*B* = −0.006, *p* = 0.847). Higher emotion regulation skills were not associated with higher empathic competences for toddlers who experienced mothers with emotion-coaching style. The total model accounted for 42% of variance in toddlers’ empathic responses, *R*^2^ = 0.42, *F*(3,308) = 15.788, *p* < 0.001, but only the two predictors significantly contributed to explain the variance (*B* = 0.200, *p* = 0.040 and B = 0.883, *p* < 0.001 for maternal emotion-coaching style and children’s emotion regulation, respectively).

## Discussion

The aim of this study was to investigate the role of individual and family factors in toddlers’ empathy. To our knowledge, this is the first study that has simultaneously explored the contributions of children’s emotion regulation, language ability, and maternal emotion style to explaining empathic responses during toddlerhood.

We obtained two main findings. First, emotion regulation, language ability, and maternal emotion-coaching style were significantly associated with children’s empathy. Second, positive emotion regulation and maternal emotion-coaching style both significantly contributed to explaining variance in empathy. Each of these findings is now discussed in detail.

### Empathy and Associations With Emotion Regulation, Vocabulary, and Maternal Emotion Socialization Style

The initial key finding of this study was that most of the research variables were associated with empathy. First of all, children who obtained higher scores for positive emotion regulation were reported by their mothers to engage in more empathic responses. This is in line with previous findings showing that greater ability to manage and modulate emotional arousal is positively associated with empathic concern and prosocial response in toddler, preschool, and school years (Panfile and Laible, 2012; Laible et al., 2014; Peterson et al., 2018). In order to show concern for other people, children need to understand that the emotions and feelings of others are separate from their own emotional experience (Hoffman, 2008; Montroy et al., 2016). They gradually develop this insight during toddlerhood, as they acquire an increasingly sophisticated understanding of their own and others’ internal states (Denham, 1998). During this stage in their development, it seems key for children to possess emotion regulation abilities that help them distinguish their own feelings from those of interlocutors and avoid being vicariously overwhelmed by the emotional experiences of others. Hence, the more children can regulate their own emotions, the more they will be able to redirect attention away from themselves and demonstrate empathic concern toward others. On the contrary, children with low regulation skills are at risk of displaying reactive and aggressive tendencies that may exacerbate externalizing problems (Peterson et al., 2018). In this study, children’s empathy was also positively associated with the breadth of their vocabulary. As documented by some authors, the relationship between language abilities and empathy is reciprocal and remains stable over time (Ensor et al., 2011; Girard et al., 2017). This of course does not mean that children are not responsive to socialization influences. Indeed, alongside the individual variables just discussed, we found that family factors were also associated with empathy. Specifically, empathic responses were significantly and positively related to maternal emotion-coaching style, consistently with past longitudinal studies involving toddlers (Brophy-Herb et al., 2011; Taylor et al., 2013). Indeed, the quality of parent-child relationships and interactions seems to be associated with empathy-related responding. When mothers predominantly accept and validate their children’s emotions, helping them deal with negative affect and identify appropriate emotion regulation strategies, this may encourage young children to be attentive toward others’ emotions and display empathy. In this regard, a significant body of literature suggests that a maternal emotion-coaching style is a protective factor for the development of social-emotional competences from the earliest years of life (see Brophy-Herb et al., 2011; Ornaghi et al., 2019). These results should be interpreted in light of the Italian cultural context, where adults generally encourage children’s emotional experiences and expressiveness (Halberstadt and Lozada, 2011; Lozada and Halberstadt, 2015). To this regard, it would be interesting to replicate this study in non-Western cultures where beliefs about emotions and educational practices may vary (Fiorilli et al., 2015).

Moreover, no significant associations emerged between a maternal emotion-dismissing style and children’s empathy. However, significant negative correlations were found between maternal emotion-dismissing style and both positive regulatory abilities and vocabulary in children, and significant and positive associations emerged between maternal emotion dismissing and poor children’s emotion regulation. Adults with this emotion style are characterized by a lack of emotional awareness and a lesser inclination to speak about emotions, and they also tend to devalue, minimize, or ignore children’s emotions (Lunkenheimer et al., 2007). This emotion socialization style has a well-documented impact on toddlers’ social-emotional development and may slow it down (Luebbe et al., 2011; Ornaghi et al., 2019). Thus, we might speculate that the relationship between maternal emotion-dismissing style and children’s empathic responses may only become apparent later in development, when advanced skill sets are demanded and deficits in regulatory abilities and vocabulary more evidently compromise the engagement in positive social interactions and peer relationships.

### The Contribution of Emotion Regulation and Maternal Emotion-Coaching Style to Accounting for Variance in Empathy

With regard to the contribution of emotion regulation and maternal emotion socialization style to explaining empathy, after controlling for age and language ability, the regression analysis showed that only positive emotion regulation and maternal emotion-coaching style significantly explained variance in empathic skills. With respect to emotion regulation, our finding is in line with the scanty existing literature on toddlers and preschoolers (Panfile and Laible, 2012; Peterson et al., 2018). Children’s ability to control their emotional arousal seems to help them redirect their attention from their own distress to that of others, which in turn elicits empathic responses. Notably, children learn to regulate their emotions thanks to their caregiver’s sensitive and contingent responsiveness. Indeed, emotion coaching by mothers was the other variable that carried significant weight in explaining toddlers’ empathy, in keeping with previous research findings (Brophy-Herb et al., 2011; Eisenberg et al., 2011; Taylor et al., 2013). This suggests that when mothers have a positive approach to emotions, they are more likely to implement socialization practices that, in turn, teach their children to accept and attend to their emotional experience, as well as to that of others, an attitude that prompts empathy.

Overall, data analyses showed an association of both maternal emotion-coaching style and children’s emotion regulation with toddlers’ empathic skills, in line with Eisenberg et al.’s (1998) heuristic model where emotion-related parenting practices contribute to children’s social and emotional competences. However, the association between maternal emotion-coaching style and children’s empathy was not moderated by emotion regulation skills. This finding suggests that other individual or family factors, which have not been taken into account in the present study, may contribute to explaining variance in toddlers’ empathy. For instance, Spinrad and Gal (2018) pointed out the importance of examining temperamental characteristics, such as children’s dispositional prosociality, as moderators/mediators that may drive early empathic responses. Similarly, there is evidence that attachment security is linked with empathic responses, in fact more secure children are reported to be higher in both emotion regulation and empathy (Panfile and Laible, 2012). More-comprehensive models would help understand which moderators contribute to toddlers’ empathic skills.

In the present study we also found that vocabulary did not significantly contribute to explaining variance in toddlers’ empathic responses. Some scholars have suggested that toddlers’ vocabulary influences the tendency to act empathically and prosocially, both directly (Ensor et al., 2011; Rhee et al., 2013; Girard et al., 2017; Conte et al., 2018) and indirectly (Ensor and Hughes, 2005; Grazzani et al., 2016a; Ornaghi et al., 2017), whereas others have identified no effect of productive language ability on children’s empathy. For example, Barnett et al. (2012) measured language skills and prosociality in children of the same age as the participants in our study. Although they identified significant associations between toddlers’ vocabulary and empathy, language skills at 24 months did not predict social competence at 36 months.

The finding in the current study may depend on the age of children. Development of the ability to regulate emotions and the influence of maternal emotion style occur very early in childhood, in fact there is evidence that newborns are able to self-regulate emotions (Calkins and Leerkes, 2011) and that caregivers’ emotion socialization and responsiveness affect children’s development from the first months of life (Morris et al., 2007; Brophy-Herb et al., 2011). The strong early association between children’s emotion regulation skills and maternal emotion socialization style may justify the fact that these factors contributed more than vocabulary to explaining variance in empathy. Indeed, there is evidence that mothers’ socialization style is strongly and positively related to children’s emotion regulation skills (e.g., Li et al., 2019; Ornaghi et al., 2019), a pattern that was confirmed by the present study. As infant research has shown, positive parent-child relationships are characterized by more intense emotional engagement that supports and guides the child in the self-regulation of emotional experiences (Gus et al., 2015). On the contrary, continuous adverse interactive experiences can undermine not only the child’s ability to modulate emotional arousal but also the development of empathic responses (Gottman et al., 1997). Since the width of vocabulary begins to increase considerably in toddler years and at this age its development is still in an early stage, language may only come to significantly influence empathy later on in children’s development. Future research should clarify this issue.

### Limitations, Future Research Directions, and Educational Implications

This paper is not without its limitations. First, we relied on a maternal report measures, with the associated risk of bias and subjectivity. In fact, the same respondent reported on her own emotion socialization style and child’s skills, likely resulting in a bias due to common shared variance. In the future, this kind of measure should be combined with more ecological instruments, such as observation grids designed to collect information about toddlers’ emotion contagion, attention to others’ feelings, and prosocial actions. Furthermore, although the contribution of mothers’ emotion-coaching style was significant, it explained only a small portion of variance in empathic skills. This may be due to the questionnaire itself (MESQ), which taps into parental emotion socialization beliefs rather than practices and asks about responses to few emotions (i.e., sadness and anger). In the future, it should be advisable to use measures that would better bring out parents’ socialization practices (e.g., observation of spontaneous mother-child interactions). A second limitation lies in the study’s cross-sectional design, which prevented us from identifying predictive relationships. Although the current results are promising, only longitudinal research can truly enhance our understanding of the contribution of intra-individual and family factors to explaining variance in toddlers’ empathy.

Despite these limitations, the present study points up the key role of both emotion regulation skills and maternal emotional style in explaining empathy in toddlerhood. Intervention studies suggest that it is possible to significantly enhance parents’ coaching responses, thereby stimulating advances in both toddlers’ (Law et al., 2014) and preschoolers’ (Havighurst et al., 2010) social and emotional development. Specifically, these programs fostering attuned, responsive, and supportive parent-child relationships have been shown to lead caregivers to engage in a higher proportion of emotion-coaching behaviors, as reflected in both self-report and observation measures, and to use more emotion-state talk when interacting with their children. Such programs also wield a protective effect by bringing about significant decreases in parental dismissing practices and child externalizing behaviors. Hence, promoting and maintaining positive parent-child relationships will be a fundamental component of future parenting programs aimed at enhancing children’s emotional and behavioral well-being. Similar programs may be implemented in early childhood education settings as well, such as nursery and kindergarten, where educators may be trained to adopt emotion-coaching responses (e.g., Ciucci et al., 2015, 2018). On the other hand, toddlers may benefit from interventions at nursery that are especially focused on enhancing their emotion regulation skills, with a view to fostering empathic responses toward others (Sprung et al., 2015; Grazzani et al., 2016a,b; Ornaghi et al., 2017).

## Data Availability Statement

The datasets generated for this study are available on request to the corresponding author.

## Ethics Statement

The studies involving human participants were reviewed and approved by the Ethics Committee of the University of Milano-Bicocca, Italy. Written informed consent to participate in this study was provided by the participants’ legal guardian/next of kin.

## Author Contributions

VO made a key contribution to designing the study, collecting, analyzing, interpreting and discussing the data, and writing the manuscript. EC made a key contribution to interpreting and discussing the data, and writing the manuscript. IG made a key contribution to interpreting and discussing the data, and revising the manuscript. All authors contributed to the article and approved the submitted version.

## Conflict of Interest

The authors declare that the research was conducted in the absence of any commercial or financial relationships that could be construed as a potential conflict of interest.
